# Differential sensitivity of the 2020 revised comprehensive diagnostic criteria and the 2019 ACR/EULAR classification criteria across IgG4-related disease phenotypes: results from a Norwegian cohort

**DOI:** 10.1186/s13075-023-03155-y

**Published:** 2023-09-05

**Authors:** Jens Vikse, Øyvind Midtvedt, Bjørg-Tilde Svanes Fevang, Torhild Garen, Øyvind Palm, Marianne Wallenius, Gunnstein Bakland, Katrine Brække Norheim, Øyvind Molberg, Anna-Maria Hoffmann-Vold

**Affiliations:** 1 Department of Clinical Science, University of Bergen, Jonas Lies Veg 87, 5021 Bergen, Norway; 2 Department of Rheumatology, Stavanger University Hospital, Stavanger, Norway; 3 Department of Rheumatology, Oslo University Hospital, Oslo, Norway; 4 Department of Rheumatology, Haukeland University Hospital, Bergen, Norway; 5 Department of Rheumatology, St. Olav University Hospital, Trondheim, Norway; 6 Department of Neuromedicine and Movement Science, Norwegian University of Science and Technology, Trondheim, Norway; 7 Department of Rheumatology, University Hospital of North Norway, Tromsø, Norway; 8 Department of Clinical Medicine, University of Oslo, Oslo, Norway; 9 Department of Rheumatology, University Hospital Zurich, University of Zurich, Zurich, Switzerland

**Keywords:** IgG4-RD, Criteria, Phenotypes

## Abstract

**Background:**

We investigated sensitivity of the 2020 Revised Comprehensive Diagnostic Criteria (RCD) and the 2019 ACR/EULAR classification criteria across the four identified IgG4-related disease (IgG4-RD) phenotypes: “Pancreato-Hepato-Biliary”, “Retroperitoneum and Aorta”, “Head and Neck-limited” and “Mikulicz’ and Systemic” in a well-characterized patient cohort.

**Methods:**

We included adult patients diagnosed with IgG4-RD after comprehensive clinical assessment at Oslo University Hospital in Norway. We assigned patients to IgG4-RD phenotypes based on pattern of organ involvement and assessed fulfillment of RCD and 2019 ACR/EULAR classification criteria. Differences between phenotype groups were analyzed using one-way ANOVA for continuous variables, and contingency tables for categorical variables.

**Results:**

The study cohort included 79 IgG4-RD patients assigned to the “Pancreato-Hepato-Biliary” (22.8%), Retroperitoneum and Aorta” (22.8%) “Head and Neck-limited” (29.1%), and “Mikulicz’ and Systemic” (25.3%) phenotype groups, respectively. While 7﻿2/79 (91.1%) patients in total fulfilled the RCD, proportion differed across phenotype groups and was lowest in the “Retroperitoneum and Aorta” group (﻿66.7%, *p* < 0.001). Among the 57 (72.2%) patients meeting the 2019 ACR/EULAR classification criteria, proportion was again lowest in the “Retroperitoneum and Aorta” group (27.8%, *p* < 0.001).

**Conclusion:**

The results from this study indicate that IgG4-RD patients having the “Retroperitoneum and Aorta” phenotype less often fulfill diagnostic criteria and classification criteria than patients with other IgG4-RD phenotypes. Accordingly, this phenotype is at risk of being systematically selected against in observational studies and randomized clinical trials, with potential implications for patients, caregivers and future definitions of IgG4-RD.

## Introduction

IgG4-related disease (IgG4-RD) is a fibroinflammatory systemic disease that can involve nearly any organ. Core features include tissue infiltration of IgG4-positive plasma cells causing tumefactive lesions and/or organomegaly, frequently accompanied by elevated serum IgG4 concentration [1].

IgG4-RD is a diagnostic challenge, owing to its heterogeneous presentations and lack of pathognomonic features. Diagnosis requires correlation of clinical, serological, radiological and/or histopathological findings [2]. Comprehensive Diagnostic Criteria (CDC) was devised in 2011 [3] and revised in 2020 (revised CDC, RCD) [4] to aid diagnosis. The CDC and RCD focus on core disease features, but their sensitivity and specificity have not been systematically evaluated [4]. Therefore, the diagnosis of IgG4-RD currently rests on expert clinical assessment.

The unsettled status of diagnostic criteria for IgG4-RD is not unexpected. It reflects that development of accurate diagnostic criteria for complex diseases with overlap to mimicking conditions is inherently challenging, as evident from the near complete absence of diagnostic criteria in rheumatology [5]. Instead, ACR and EULAR have invested major resources in the development of classification criteria for research purposes [5]. In general, classification criteria aim to select homogenous cases from patient cohorts clinically diagnosed with the disease in question. As this purpose, by definition, requires high specificity, a potential weakness of classification criteria is that they may need to sacrifice sensitivity to optimize specificity. Though not intended, low sensitivity may introduce biases, including skewed representation of disease phenotypes. If low sensitivity of classification criteria skews phenotype distribution, research output will suffer from the same bias.

The 2019 ACR/EULAR IgG4-RD classification criteria were developed by an international expert group. In the two separate validation cohorts, the reported sensitivities of the criteria were 85.5% and 82.0%, respectively, while specificities were 99.2% and 97.8% [2].

Following publication of the 2019 ACR/EULAR classification criteria, Wallace et al. used data from the validation cohorts to identify four distinct clinical phenotypes of IgG4-RD with different patterns of organ involvement: (i) “Pancreato-Hepato-Biliary”; (ii) “Retroperitoneum and Aorta”; (iii) “Head and Neck-limited” and (iv) “Mikulicz’ and Systemic” [6]. Importantly, in addition to different organ involvement, the phenotypes differed in demographic features and serum IgG4 concentrations, indicating biological differences which may impact disease course. To date, there are no results from independent IgG4-RD cohorts showing how well the RCD and the 2019 ACR/EULAR classification criteria perform across the four phenotypes.

Here, we aimed to assess sensitivity of the RCD and the 2019 ACR/EULAR classification criteria across the four phenotypes. We included a well-characterized Norwegian cohort of patients with IgG4-RD diagnosed by expert clinical assessment, stratified by phenotype, and assessed criteria performance. As our study cohort did not include patients diagnosed with mimicking conditions, we were not able to assess the specificity of the criteria.

## Methods

At the Department of Rheumatology at Oslo University Hospital (OUH) we consecutively include consenting adult patients (≥ 18 years) diagnosed with IgG4-RD by expert clinical assessment in the Norwegian systemic connective tissue disease and vasculitis registry (NOSVAR) [7]. For this study, we included IgG4-RD patients from NOSVAR diagnosed from 2001–2022. Data was retrieved from NOSVAR and the electronic medical journal.

Elevated serum IgG4 levels were defined as > 1.35 g/L for the CDC and RCD criteria [3, 4], and > 2.01 g/L (upper limit of normal range at the OUH laboratory) for the 2019 ACR/EULAR classification criteria [2], as per the criteria’s definitions.

Organ involvement was determined by clinical, histopathological and/or radiological findings, where other causes were deemed unlikely. Multi-organ involvement was defined as ≥ 2 involved organs. Two rheumatologists (JV, ØMi) assessed fulfilment of the CDC, RCD and 2019 ACR/EULAR classification criteria, and assigned patients to one out of four phenotypes based on pattern of organ involvement [6].

Written informed consent was given for the included IgG4-RD patients as requirement for inclusion in NOSVAR. The study was conducted in compliance with the Helsinki Declaration and approved by the regional ethics committee (REK #342136).

### Assessment of CDC and RCD

Both the CDC and RCD include three variables: (i) clinical and radiological findings suggestive of IgG4-RD; (ii) elevated serum IgG4 level (defined as > 1.35 g/L); and (iii) compatible histopathological findings [3, 4]. According to the CDC and RCD statement, patients were designated as “definite” (i + ii + iii), “probable” (i + iii) or “possible” (i + ii) IgG4-RD cases. Fulfilment of the histopathological domain of CDC requires both (a) lymphoplasmacytic infiltration and fibrosis and (b) > 10 IgG4-positive (IgG4 +) plasma cells per high power field (hpf) and ratio of IgG4 + /IgG4 + plasma cells > 0.40 [3]. The histopathological domain of RCD includes the same two variables, but also (c) typical tissue fibrosis, particularly storiform fibrosis, or obliterative phlebitis, and fulfilment requires at least two of three [(a), (b) and/or (c)] [4]. Exclusion criteria for CDC and RCD are listed in the original documents [3, 4] and include mimicking conditions such as granulomatosis with polyangiitis and eosinophilic granulomatosis with polyangiitis.

### Assessment of 2019 ACR/EULAR classification criteria

The 2019 ACR/EULAR classification criteria employ a three-step approach, which includes (i) an obligatory entry criterion (involvement of a typical organ with compatible clinical and/or histopathological features); (ii) a set of exclusion criteria; and (iii) a list of classification items with weighted scores assigned to various clinical, serological, and histopathological features. Following exclusion of mimickers, we classified patients as IgG4-RD cases if they (i) met the entry criterion, (ii) had no exclusion criteria and (iii) scored ≥ 20 points by the defined classification items [2].

### Outcome measures

In this cohort of well-characterized patients diagnosed with IgG4-RD based on expert clinical assessment, we aimed to describe, both on a group and phenotypic level:

- Fulfilment of CDC, RCD and 2019 ACR/EULAR classification criteria
- Reasons for failure to fulfil the criteria

### Statistics

Descriptive statistics were applied, using IBM SPSS version 26 for Windows (Armonk, NY: IBM Corp.). Continuous variables are reported as means and standard deviations, and between-group differences analyzed using one-way ANOVA. Categorical variables are reported as absolute number and percentage, and between-group differences analyzed using contingency tables.

## Results

### Baseline characteristics, phenotypes, and fulfilment of criteria

The IgG4 study cohort included 79 patients (Table 1). In the “Head and Neck-limited” group, patients were younger (*p* = 0.002), more often female (*p* = 0.024), and demonstrated a trend toward more non-white patients. The “Retroperitoneum and Aorta” group had the highest mean CRP (*p* < 0.001) and ESR (*p* = 0.001) and was characterized by the lowest mean serum IgG4 concentration, less frequent multi-organ disease (*p* = 0.03), and fewer biopsies (*p* < 0.001). The “Mikulicz’ and Systemic” group had the highest mean serum IgG4 concentration and mean number of involved organs (*p* < 0.001 for both).

**Table 1:** Baseline characteristics and phenotypic distribution of the IgG4-RD study cohort

	All	Pancreato-Hepato-Biliary	Retroperitoneum and Aorta	Head and Neck-Limited	Mikulicz’ and Systemic	*p*-value
N (%)	79 (100)	18 (22.8)	18 (22.8)	23 (29.1)	20 (25.3)	
Male subjects, n (%)	53 (67.1)	15 (83.3)	12 (66.7)	10 (43.5)	16 (80.0)	0.024
White, n (%)	63 (79.7)	17 (94.4)	15 (83.3)	15 (65.2)	16 (80.0)	0.135
Age at diagnosis, years (SD)	57.8 (14.2)	62.3 (14.9)	63.8 (8.8)	48.8 (16.0)	58.6 (10.3)	0.002
Serum IgG4, g/L (SD) (*n* = 73) ^a^	6.06 (6.02)	5.35 (4.66)	2.5 (1.6)	4.6 (4.4)	11.6 (7.7)	< 0.001
Elevated baseline serum IgG4, n (%) (*n* = 76) ^a^	59/76 (77.6)	13/18 (72.2)	11/16 (68.8)	17/22 (77.3)	18 (90.0)	0.425
CRP, mg/dL (SD)	12.9 (25.6)	4.6 (6.6)	37.0 (44.7)	8.3 (15.6)	6.0 (7.5)	< 0.001
ESR, mm/h (SD)	32.3 (29.6)	17.7 (11.1)	56.4 (35.4)	23.3 (25.7)	32.6 (25.9)	0.001
Biopsy, n (%)﻿ ^b^	62 (78.5)	17 (94.4)	6 (33.3)	22 (95.7)	17 (85.0)	< 0.001
Number of involved organs (SD)	3.7 (2.1)	3.3 (1.5)	2.2 (1.6)	2.9 (1.4)	6.2 (1.5)	< 0.001
≥ 2 involved organs, n (%)	68 (86.1)	17 (94.4)	11 (61.1)	20 (87.0)	20 (100)	0.003
﻿RCD, (%﻿)						< 0.001
Any	72 (91.1)	18 (100)	12 (66.7)	23 (100)	19 (95.0)	
Definite	39 (49.4)	13 (72.2)	2 (11.1)	11 (47.8)	13 (65.0)	
Probable	14 (17.7)	4 (22.2)	2 (11.1)	7 (30.4)	1 (5.0)	
Possible	19 (24.1)	1 (5.6)	8 (44.4)	5 (21.7)	5 (25.0)	
ACR/EULAR classification criteria, n (%)	57 (72.2)	18 (100)	5 (27.8)	14 (60.9)	20 (100)	< 0.001

Continuous variables were analyzed by one-way ANOVA. Categorical variables were analyzed by contingency tables

*CRP* C-reactive protein, *ESR* erythrocyte sedimentation rate, *RCD* revised comprehensive diagnostic criteria

^a^ Some patients did not measure serum IgG4 (s-IgG4) before initiation of immunosuppressive therapy. These were considered to have elevated baseline s-IgG4 if they had elevated levels after initiation of treatment; or excluded if they had normal s-IgG4 after initiation of treatment. Elevated s-IgG4 = above the upper limit of normal in the Oslo University Hospital laboratory assay (≥ 2,01 g/L)

^b^ Excluding fine needle aspiration

In total, 72 patients (91.1%) fulfilled the CDC and RCD. Discrepancy between CDC and RCD only occurred twice: two patients deemed “possible” IgG4-RD by CDC were considered “definite” by RCD. This discrepancy related to the histopathological domain of these criteria. Both patients had dense lymphoplasmacytic infiltrate with fibrosis, and > 10 IgG4 + plasma cells per hpf. The tissue IgG4 + /IgG + plasma cell ratio was < 0.40 (hence, “possible” by CDC), but there was evidence of storiform fibrosis and obliterative phlebitis (hence, “definite” by RCD). Given these minor differences, we decided to focus on RCD for all further analyses. Fulfilment of RCD was lower in the “Retroperitoneum and Aorta” group﻿ (66.7%) than in the remaining groups: 100% in “Pancreato-Hepato-Biliary”, 100% in “Head and Neck-limited” and 95.0% in “Mikulicz’ and Systemic” phenotype. The between-group difference was statistically significant (*p* < 0.001).

Fifty-seven patients (72.2%) in the IgG4-RD cohort fulfilled the 2019 ACR/EULAR classification criteria, with 100% meeting the criteria in both the “Pancreato-Hepato-Biliary” and “Mikulicz’ and Systemic” groups. The percentage of patients fulfilling the classification criteria was lower in the “Retroperitoneum and Aorta” group (27.8%) and the “Head and Neck-limited” group (60.9%) (*p* < 0.001).

Among the 22 patients who did not fulfill the 2019 ACR/EULAR classification criteria, 13 (59.0%) had elevated baseline serum IgG4 (> 2.01 g/L), and 11 (50.0%) had performed biopsy, excluding fine needle aspiration.

### Reasons for failure to fulfil the 2019 ACR/EULAR classification criteria

**Fig. 1:**
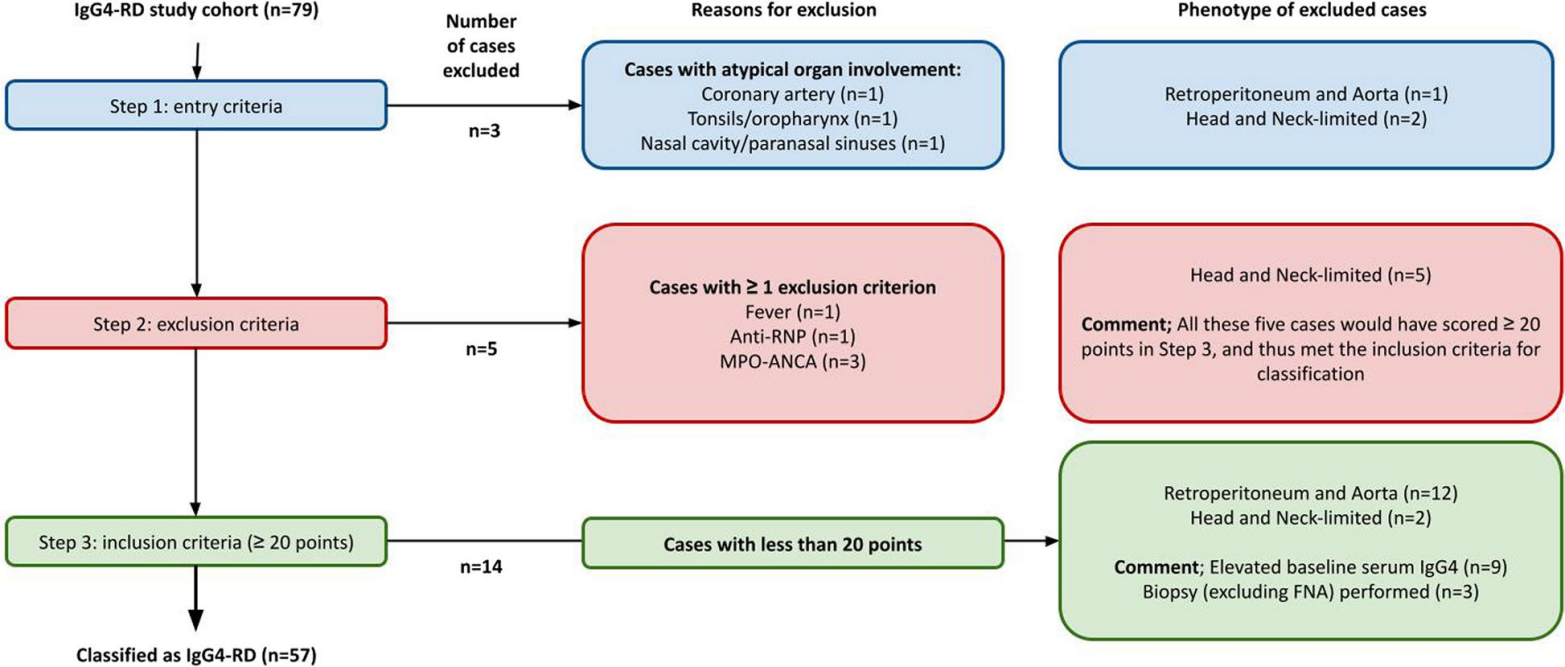
Legend: F﻿ulfillment of the 2019 ACR/EULAR classification criteria in the Norwegian IgG4-RD cohort

The reasons why the 22 patients did not meet the 2019 ACR/EULAR classification criteria are summarized in Fig. 1 and Tables 2 and 3. Reasons for failure to fulfil the criteria included (i) failure to meet the inclusion criterion (*n* = 3), (ii) fulfilment of one or more exclusion criteria (*n* = 5) or (iii) failure to achieve the required 20 points (*n* = 14).

**Table 2:** Patients with the “Retroperitoneum and Aorta” phenotype, who failed to fulfil the 2019 ACR/EULAR classification criteria

Case	Features	Histopathological findings	s-IgG4 (g/L) (ref > 1.35 for RCD, > 2.01 for 2019 ACR/EULAR classification criteria)	RCD	2019 ACR/EULAR classification criteria
1	Abdominal pain. RPF in a typical distribution. No other manifestations	Not performed	5.08	Possible IgG4-RD	Fulfils entry criterion. No exclusion criteria Weighted score = 14 • s-IgG4 = 6 • RPF = 8
2	Abdominal pain. RPF in a typical distribution. No other manifestations	Not performed	2.15	Possible IgG4-RD	Fulfils entry criterion. No exclusion criteria Weighted score = 12 • s-IgG4 = 4 • RPF = 8
3	Abdominal pain. RPF in a typical distribution. No other manifestations	Inconclusive fine needle aspiration	0.63	Not fulfilled	Fulfils entry criterion. No exclusion criteria Weighted score = 8 • RPF = 8
4	Abdominal pain. iAAA and RPF in a typical distribution. No other manifestations	Not performed	2.14	Possible IgG4-RD	Fulfils entry criterion. No exclusion criteria Weighted score = 12 • s-IgG4 = 4 • RPF = 8
5	Abdominal pain. RPF in a typical distribution. No other manifestations	Not performed	0.50	Not fulfilled	Fulfils entry criterion. No exclusion criteria Weighted score = 8 • RPF = 8
6	Abdominal pain. RPF in a typical distribution. No other manifestations	Not performed	2.90	Possible IgG4-RD	Fulfils entry criterion. No exclusion criteria Weighted score = 12 • s-IgG4 = 4 • RPF = 8
7	Renal failure and hydronephrosis. RPF in a typical distribution. No other manifestation	Not performed	3.20	Possible IgG4-RD	Fulfils entry criterion. No exclusion criteria Weighted score = 12 • s-IgG4 = 4 • RPF = 8
8	Abdominal pain. RPF in a typical distribution. FDG-uptake in non-enlarged bilateral parotids. No other manifestations	Parotid gland biopsy: inconclusive	0.80	Not fulfilled	Fulfils entry criterion. No exclusion criteria Weighted score = 14 • One set glands = 6 • RPF = 8
9	Hydronephrosis. RPF in a typical distribution. No other manifestations	Not performed	2.87	Possible IgG4-RD	Fulfils entry criterion. No exclusion criteria Weighted score = 12 • s-IgG4 = 4 • RPF = 8
10	Abdominal pain and renal failure. RPF in a typical distribution. No other manifestations	Not performed	2.00	Possible IgG4-RD	Fulfils entry criterion. No exclusion criteria Weighted score = 8 • ﻿RPF = 8
11	Long standing, severe asthma, rhinosinusitis and nasal polyposis. 15 years prior to IgG4-RD diagnosis: eosinophilic pneumonia with blood hypereosinophilia. Mild polyneuropathy. No other EGPA-manifestations. Negative ANCA. Nasal polyps demonstrated subacute inflammation with tissue eosinophilia, without evidence of vasculitis or granulomas. Treated with mepolizumab, which facilitated discontinuation of glucocorticoids. Two years prior to IgG4-RD diagnosis: AAA (without evidence of RPF). At time of diagnosis: large right coronary artery aneurysm (CAA). No other organ manifestations. Deemed as coexisting EGPA and IgG4-RD, with the latter accounting for the CAA. The rational was (i) the remaining EGPA manifestations were well controlled; (ii) CAA is an increasingly recognized manifestation of IgG4-RD; and (iii) previous reports of coexistence of IgG4-RD and AAV. However, we acknowledge the diagnostic uncertainty	Resected coronary artery aneurysm: LPC infiltrate and fibrosis (without storiform appearance), 72 IgG4 + PC/hpf and IgG4/IgG-ratio 0.55. Mild tissue eosinophilia	3.20	Not fulfilled (Exclusion criterion: EGPA)	Does not fulfil entry criterion (coronary artery not listed as a typical organ). No exclusion criteria Weighted score = 22 • Histology = 4 • IHC = 14 • s﻿-IgG4 = 4
12	Abdominal pain and hydronephrosis. RPF in a typical distribution, in combination with aortitis and iAAA. No other manifestations	Not performed	Not measured prior to glucocorticoids. < 1.35 following treatment initiation	Not fulfilled	Fulfils entry criterion. No exclusion criteria Weighted score = 8 • RPF = 8
13	Abdominal pain and hydronephrosis. RPF in a typical distribution. No other manifestations	Not performed	0.74	Not fulfilled	Fulfils entry criterion. No exclusion criteria Weighted score = 8 • RPF = 8

*AAA* abdominal aortic aneurysm,﻿ *AAV* ANCA associated vasculitis *ANCA* anti-neutrophilic cytoplasmic antibody, *CAA* coronary artery aneurysm, *EGPA* eosinophilic granulomatosis with polyangiitis, *hpf* high power field, *iAAA* inflammatory abdominal aortic aneurysm, *IgG4* + IgG4 positive, *IHC* immunohistochemistry, *LPC* lymphoplasmacytic, *PC* plasma cells, *RPF* retroperitoneal fibrosis, *s-IgG4* serum IgG4

**Table 3:** Patients with the “Head and Neck-Limited” phenotype, who failed to fulfil the 2019 ACR/EULAR classification criteria

Case	Features	Histopathological findings	s-IgG4 (g/L) (ref > 1.35 for RCD, > 2.01 for 2019 ACR/EULAR classification criteria)	RCD	2019 ACR/EULAR classification criteria
1	Two years prior to IgG4-RD diagnosis: diagnosis of MPA based on petechiae, glomerulonephritis (biopsy proven) and positive MPO-ANCA. Treated with RTX (1A and 1B), followed by AZA maintenance. At time of IgG4-RD diagnosis (under maintenance therapy with AZA monotherapy): left lacrimal gland enlargement. No other manifestations. Deemed to be coexisting MPA and IgG4-RD, with the latter accounting for the dacryoadenitis. The rational was (i) the other vasculitic manifestations were well controlled; and (ii) the previous reports of coexistence of AAV and IgG4-RD. However, we acknowledge the diagnostic uncertainty	Lacrimal gland: LPC infiltrate and storiform fibrosis. > 50 IgG4 + PC/hpf with IgG4/IgG ratio > 0.40	0.81	Probable IgG4-RD	Fulfils entry criterion. Fulfils exclusion criterion (MPO-ANCA) Weighted score = 27 • Histology = 13 • IHC = 14
2	Sinusitis, cough and low-grade fever. Destructive process in sphenoid sinus, lymphadenopathy and lung changes (ground glass opacities and small nodules). ANCA negative. Biopsy without evidence of AAV or malignancy	Sphenoid sinus: LPC infiltrate and fibrosis (without storiform appearance). Abundant IgG4 + PC/hpf (unable to quantitate), with IgG4/IgG ratio > 0.40	3.43	Definite IgG4-RD	Fulfils entry criterion. Fulfils exclusion criterion (fever) Weighted score = 22 • Histology = 4 • IHC = 14 • s-IgG4 = 4
3	Inflammatory mass in nasal septum. No other definite manifestations (albeit possible idiopathic pancreatitis 10 years prior). ANCA negative. Biopsy without evidence of AAV or malignancy	Nasal septum: LPC infiltrate and fibrosis (without storiform appearance). Abundant IgG4 + PC/hpf (unable to quantitate), with IgG4/IgG ratio > 0,3	2.08	Definite IgG4-RD	Does not fulfil entry criterion (nasal septum not a typical organ). No exclusion criteria Weighted score = 15 • Histology = 4 • IHC = 7 • s-IgG4 = 4
4	Chronic rhinosinusitis and arthralgia. Negative ANCA. FDG-uptake in tonsils, oropharynx, and bilateral parotid and submandibular glands. No other manifestations. Positive anti-RNP, but no arthritis or other manifestations suggestive of MCTD or other connective tissue disease	Waldeyers ring: LPC infiltrate. 88 IgG4 + PC/hpf, with IgG4/IgG ratio > 0.4	4.30	Definite IgG4-RD	Fulfils entry criterion. Fulfils exclusion criterion (anti-RNP) Weighted score = 38 • Histology = 4 • IHC = 14 • s-IgG4 = 6 • Two sets of glands = 14
5	Right lacrimal gland enlargement. FDG-uptake in bilateral parotid and submandibular glands. No other manifestations. Positive MPO-ANCA, but no other manifestations consistent with AAV. As there was no evidence of vasculitis or extraglandular vasculitic manifestations, we believe that the presentation was more consistent with IgG4-RD than AAV	Lacrimal gland: LPC infiltrate. > 100 IgG4 + PC/hpf, with IgG4/IgG ratio > 0.4	2.30	Definite IgG4-RD	Fulfils entry criterion. Fulfils exclusion criterion (MPO-ANCA) Weighted score = 36 • Histology = 4 • IHC = 14 • s-IgG4 = 4 • Two sets of glands = 14
6	Lacrimal gland enlargement. FDG-uptake in bilateral parotid glands. No other manifestations	Lacrimal glands: inconclusive	3.17	Possible IgG4-RD	Fulfils entry criterion. No exclusion criteria Weighted score = 18 • s-IgG4 = 4 • Two sets of glands = 14
7	Orbital pseudotumor and ipsilateral lacrimal gland enlargement. FDG uptake in bilateral lacrimal and submandibular glands. No other manifestations. Positive MPO-ANCA and microscopic hematuria, with renal biopsy consistent with low-grade AAV (without evidence of IgG4-related kidney disease). Considered to be coexisting MPA and IgG4-RD, with the latter accounting for the head and neck manifestations. However, we acknowledge the diagnostic uncertainty	Lacrimal glands: LPC infiltrate and fibrosis (without storiform appearance). > 90 IgG4 + PC/hpf, unable to estimate ratio	1.30	Probable IgG4-RD	Fulfils entry criterion. Fulfils exclusion criterion (MPO-ANCA) Weighted score = 25 • Infiltrate = 4 • IHC = 7 • Two sets of glands = 14
8	Right submandibular gland enlargement and lymphadenopathy. FDG-uptake in right parotid and submandibular glands. No other manifestations	Lymph node: LPC infiltrate. > 100 IgG4 + PC/hpf and IgG4/IgG ratio 0.80	6.70	Possible IgG4-RD (lymph node biopsy not considered)	Fulfils entry criterion. No exclusion criteria Weighted score = 10 • Histology = 4 • s-IgG4 = 6 (lymph node biopsy not used for IHC scoring)
﻿9	Pharyngeal fullness and low-grade fever. FDG uptake in oropharynx and lymph nodes. No other manifestations	Waldeyers ring: LPC infiltrate and fibrosis (without storiform appearance). Abundant IgG4 + PC/hpf (uanable to quantify), with IgG4/IgG ratio > 0.50	3.40	Definite IgG4-RD	Does not fulfil entry criterion (oropharynx not a typical organ). Fulfils exclusion criterion (fever) Weighed score = 22 • Histology = 4 • IHC = 14 • s-IgG4 = 4

*AAV* ANCA associated vasculitis, *ANCA* anti-neutrophilic cytoplasmic antibody, *AZA* azathioprine, *FDG* fluorodeoxyglucose, *hpf* high power field, *ICH* immunohistochemistry, *IgG4* + IgG4 positive, *LPC* lymphoplasmacytic, *MCTD* mixed connective tissue disease, *MPA* microscopic polyangiitis, *MPO* myeloperoxidase, *PC* plasma cells, *RTX* rituximab, *s-IgG4* serum IgG4

Of the 13 patients in the “Retroperitoneum and Aorta” group who failed to fulfil the 2019 ACR/EULAR classification criteria (Table 2), 1 had isolated coronary artery involvement, while the remaining 12 had retroperitoneal fibrosis in a typical distribution (i.e., anterolateral (or circumferential) fibrosis involving the infrarenal aorta, often extending to the iliac arteries). In all the latter 12 cases, the reason for failure to fulfil the classification criteria was the inability to achieve the required 20 points in the final domain of the criteria. Of these 12 cases, (i) 11 patients (91.7%) had retroperitoneal fibrosis (with or without concomitant aortitis and/or inflammatory abdominal aortic aneurysm) as the only manifestation of the disease; (ii) 6 patients (50.0%) had elevated serum IgG4 (> 2.01 g/L), and (iii) none had a representative biopsy.

Of the 9 patients in the “Head and Neck-limited” group who failed to fulfil the 2019 ACR/EULAR classification criteria (Table 3), the clinical presentations and reasons for failure to achieve the criteria were more diverse than in the “Retroperitoneum and Aorta” group. Biopsy had been performed in all 9 cases, 7 patients (77.8%) had elevated serum IgG4 concentration (> 2.01 g/L), and 8 patients (88.9%) had multi-organ involvement. Two patients (22.2%) failed to fulfil the entry criterion (with disease limited to oropharynx and nasal septum, respectively), but were presumed to have IgG4-RD based on histopathological findings, serum IgG4 concentrations, and lack of a clear and definite alternative cause. Five (55.6%) fulfilled one or more exclusion criteria: fever (*n* = 1), positive anti-RNP (*n* = 1) or positive MPO-ANCA (*n* = 3). In the MPO-ANCA positive group, 2 patients were presumed to have coexisting IgG4-RD and microscopic polyangiitis. Of the 7 patients who failed to fulfil the entry criterion and/or fulfilled an exclusion criterion, 5 (71.4%) achieved the required 20 points in the subsequent domain of the classification criteria.

### Cases of discrepancy between RCD and the 2019 ACR/EULAR classification criteria fulfilment

**Fig. 2:**
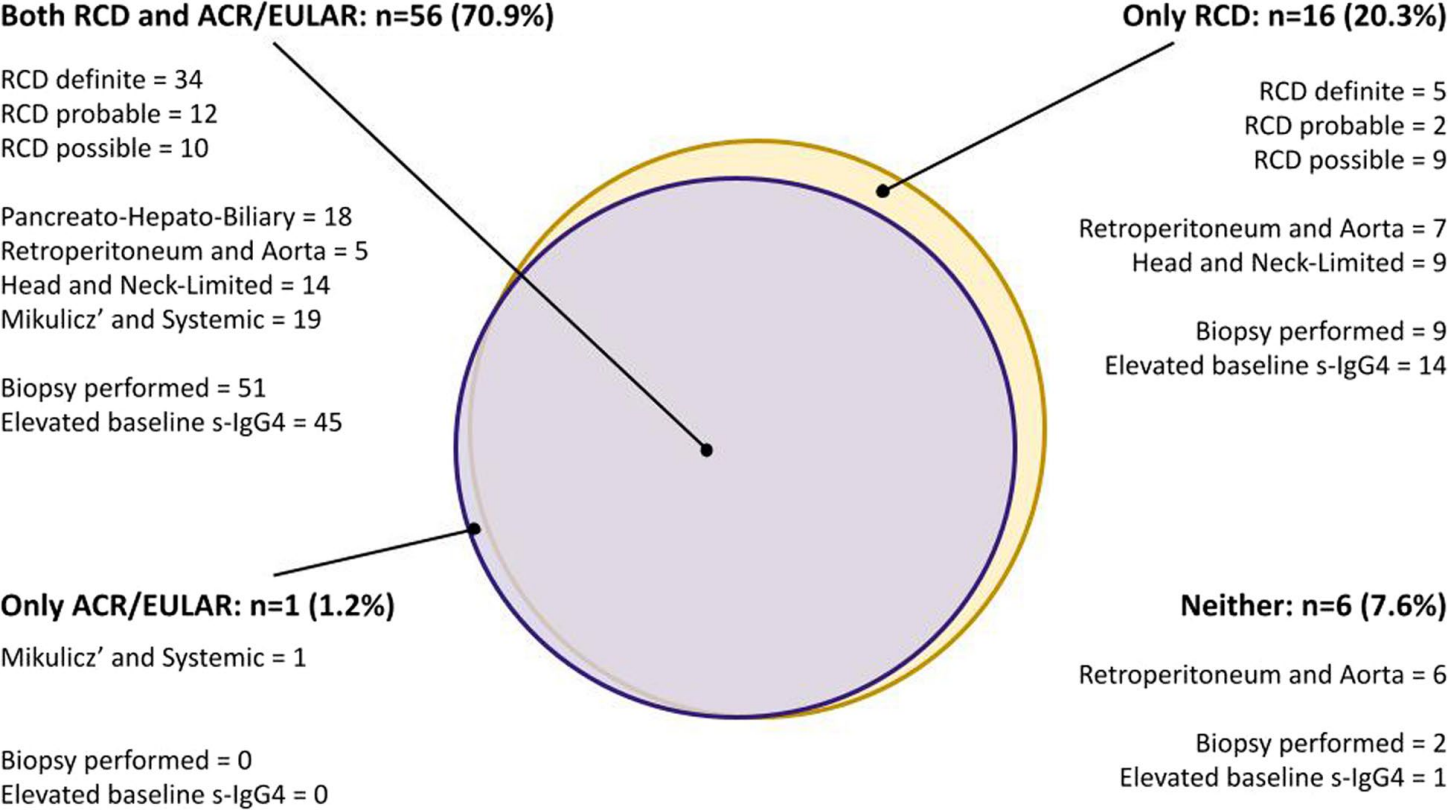
Legend﻿: Discrepancy between fulfilment of the RCD and 2019 ACR/EULAR classification criteria

Among the 22 patients who did not fulfil the 2019 ACR/EULAR classification criteria, 16 (72.7%) fulfilled the ﻿RCD, with 5, 2 and 9 patients considered “definite”, “probable” and “possible” IgG4-RD, respectively (Fig. 2). Among the 7 patients who did not fulfil RCD, one fulfilled the 2019 ACR/EULAR classification criteria. This was a patient with “Mikulicz’ and Systemic” phenotype, with characteristic and extensive multiorgan involvement and normal serum IgG4 level, where biopsy was deemed unnecessary for diagnosis.

As the current study population did not include patients diagnosed with mimicking conditions, we were not able to calculate the specificity of the criteria.

## Discussion

The performance of diagnostic and classification criteria of IgG4-RD across phenotypes is not well studied. Here, we addressed this issue using data from a well-characterized Norwegian cohort diagnosed with IgG4-RD at a tertiary referral center. The key finding in this study is low sensitivity of the 2019 ACR/EULAR classification criteria for the “Retroperitoneum and Aorta”, and “Head and Neck-limited” phenotypes of IgG4-RD. Additionally, we found that a lower proportion of patients with the “Retroperitoneum and Aorta” phenotype met the RCD compared to the other phenotyopes.

To our knowledge, our study is the first to describe fulfilment of RCD and 2019 ACR/EULAR classification criteria across the four phenotypes, highlighting potentially important differences across phenotypes. Fulfilment of classification criteria is usually a prerequisite for inclusion in studies in the field of rheumatology. Hence, the subset of patients fulfilling such criteria largely shape our understanding of a disease over time [5]. Importantly, if classification criteria do not fully capture distinct clinical phenotypes which constitute a substantial proportion of patients *and* differ in clinically important features (such as prognosis), the net result may be lost opportunities for treatment of individual patients, and skewed apprehension of disease features.

Our cohort demonstrated similar disease characteristics and phenotypic distribution as the multinational phenotype derivation cohort [6], and most patients fulfilled RCD. Despite this, only a proportion of patients in the “Retroperitoneum and Aorta” (27.8%) and “Head and Neck-limited” (60.9%) phenotypes fulfilled the 2019 ACR/EULAR classification criteria. This contrasts the findings in the phenotype derivation cohort, where the fulfilment of 2019 ACR/EULAR classification criteria in these two groups were 77% and 84%, respectively [6]. The reasons for the lower sensitivity in our cohort is not clear. It may reflect differences in case selection, possibly reflecting differences in assessment of retroperitoneal fibrosis (biopsy versus imaging). Also, it may reflect disease expression, i.e., Norwegian patients in “Retroperitoneum and Aorta” and “Head and Neck-limited” group could potentially have fewer additional manifestations and/or lower serum IgG4 than other cohorts, limiting their accrual of additional points in the classification criteria. Alternatively, one could argue that some patients in our cohort were misdiagnosed as IgG4-RD. In patients with retroperitoneal fibrosis with no other organ manifestations, normal serum IgG4, and no (conclusive) biopsy, a presumptive clinical diagnosis of possible IgG4-RD was made based on demography and radiological findings (i.e., distribution of the fibrosis), if other causes were deemed less likely, albeit with the recognition that distinction between IgG4-RD and “idiopathic retroperitoneal fibrosis” in such scenarios is difficult. The diagnosis of IgG4-RD can also be debated in some of the patients in the “Head and Neck-limited” phenotype. In general, we base the diagnosis on compatible clinical presentation (slowly progressive, painless tumefactive lesion(s) or gross organomegaly), with compatible histopathological findings, frequently accompanied by elevated serum IgG4, and absence of a definite alternative cause. While patients with overlapping features of ANCA-associated vasculitis (AAV) and IgG4-RD represent a diagnostic challenge, we considered the three patients included in this study to have coexisting AAV and IgG4-RD.

Considering the inherent ambiguity when diagnosing a complex and heterogenous disease, we chose to describe the patients not fulfilling the 2019 ACR/EULAR classification criteria, for transparency and to allow recalculation based on alternative interpretations by the readers.

The most common reason for not fulfilling the 2019 ACR/EULAR classification criteria in our IgG4-RD cohort was inability to achieve the required 20 points in the final step of the criteria [2]. It is possible that this relates to the low numeric weight assigned to typical manifestations in both the “Retroperitoneum and Aorta” and “Head and Neck-limited” phenotypes. For instance, retroperitoneal fibrosis in a typical distribution, a finding highly suggestive of IgG4-RD, yields only 8 points [2]. These patients frequently have normal or only mildly elevated serum IgG4 concentration, no other organ involvement, and are often poor candidates for biopsy due to the periaortic disease distribution [8]. This was also demonstrated in our study, with the “Retroperitoneum and Aorta” group having the lowest mean serum IgG4 level, fewer involved organs, and rarely having undergone biopsy. Similarly, orbital pseudotumor, a typical manifestation of the “Head and Neck-limited” group, does not yield any points in the classification criteria [2].

Importantly, clinical experience indicates that the “Retroperitoneum and Aorta” and “Head and Neck-limited” phenotypes are more treatment refractory than the remaining groups [8]. Taken together, these observations may indicate that the 2019 ACR/EULAR classification criteria could disfavor subsets of IgG4-RD patients with more treatment resistant disease.

As we did not have access to patients with mimicking conditions in this study, we were unable to calculate the specificity of any criteria. It is reasonable to assume that RCD has a low specificity for IgG4-RD, as it focuses on largely nonspecific features of the disease. This is particularly true for cases designated as “possible” IgG4-RD, which largely rests on elevated serum IgG4, a finding seen in many inflammatory conditions. Accordingly, we do not suggest the superiority of these criteria, nor do we support favoring their use to identify patients for clinical trials. Rather, the main finding in our study is the potential limitation of the 2019 ACR/EULAR classification criteria for certain phenotypes, which may have implications for future research. Whether increasing the weighed score assigned to “typical” retroperitoneal fibrosis and/or including orbital pseudotumor as a weighted manifestation alleviate this shortcoming without significantly sacrificing specificity is unclear but warrants further discussion. We encourage further research to evaluate the specificity of the criteria in large cohorts that include patients diagnosed with mimicking conditions.

The strength of our study is a well-described cohort followed at a tertiary referral center with rheumatologists, pathologists, radiologists, and other specialists experienced in IgG4-RD. Furthermore, the work-up included advanced imaging, including ^18^FDG PET/CT in many patients. Hence, it seems unlikely that the failure to achieve the required 20 points reflects inability to capture additional, mild and/or asymptomatic disease manifestations.

The limitations of this study include its single center design with partly retrospectively collected data and predominantly White patients. Another limitation is the lack of baseline (pre-treatment) serum IgG4 in some patients, the fact that some patients did not have a biopsy performed, and the inherent diagnostic ambiguity in this field.

## Conclusion

Our study demonstrated that the 2019 ACR/EULAR classification criteria did not capture most patients with the “Retroperitoneum and Aorta” and “Head and Neck-limited” phenotypes of IgG4-RD. Hence, through a lower ability to capture these subgroups, results from studies based on these criteria, may not be representative for the whole disease population.

## Data Availability

The datasets used during the currents study are available from the corresponding author on reasonable request.

## Abbreviations

AAA: Abdominal aortic aneurysm
AAV: ANCA associated vasculitis
ACR: American College of Rheumatology
ANCA: Anti neutrophilic cytoplasmic antibody
AZA: Azathioprine
CDC: Comprehensive diagnostic criteria
EGPA: Eosinophilic granulomatosis with polyangiitis
EULAR: European Alliance of Associations for Rheumatology
FDG: Fluorodeoxyglucose
hpf: High power field
iAAA: Inflammatory abdominal aortic aneurysm
IgG4-RD: IgG4-related disease
IHC: Immunohistochemistry
LPC: Lymphoplasmacytic
MCTD: Mixed connective tissue disease
MPA: Microscopic polyangiitis
MPO: Myeloperoxidase
PC: Plasma cell
RCD: Revised comprehensive diagnostic criteria
RPF: Retroperitoneal fibrosis
RTX: Rituximab
s-IgG4: Serum IgG4

## References

[1] Stone JH, Zen Y, Deshpande V (2012). IgG4-related disease. N Engl J Med.

[2] Wallace ZS, Naden RP, Chari S (2020). The 2019 American college of rheumatology/European league against rheumatism classification criteria for igg4-related disease. Ann Rheum Dis.

[3] Umehara H, Okazaki K, Masaki Y (2012). Comprehensive diagnostic criteria for IgG4-related disease (IgG4-RD), 2011. Mod Rheumatol.

[4] Umehara H, Okazaki K, Kawa S (2021). The 2020 revised comprehensive diagnostic (RCD) criteria for IgG4-RD. Mod Rheumatol.

[5] Aggarwal R, Ringold S, Khanna D (2015). Distinctions between diagnostic and classification criteria?. Arthritis Care Res (Hoboken).

[6] Wallace ZS, Zhang Y, Perugino CA (2019). Clinical phenotypes of IgG4-related disease: an analysis of two international cross-sectional cohorts. Ann Rheum Dis.

[7] Garen T, Lerang K, Hoffmann-Vold AM (2019). Mortality and causes of death across the systemic connective tissue diseases and the primary systemic vasculitides. Rheumatology (Oxford).

[8] Lanzillotta M, Mancuso G, Della-Torre E (2020). Advances in the diagnosis and management of IgG4 related disease. BMJ.

